# Characteristics, Outcome and Prognostic Factors of Patients with Emergency Department Cardiac Arrest: A 14-Year Retrospective Study

**DOI:** 10.3390/jcm13164708

**Published:** 2024-08-11

**Authors:** Jacopo Davide Giamello, Salvatore D’Agnano, Giulia Paglietta, Chiara Bertone, Alice Bruno, Gianpiero Martini, Alessia Poggi, Andrea Sciolla, Giuseppe Lauria

**Affiliations:** 1 School of Emergency Medicine, University of Turin, 10100 Turin, Italy; 2 Department of Emergency Medicine, Santa Croce e Carle Hospital, 12100 Cuneo, Italy

**Keywords:** emergency department, cardiac arrest, in-hospital cardiac arrest

## Abstract

**Introduction**: Cardiac arrests are traditionally classified according to the setting in which they occur, including out-of-hospital cardiac arrest (OHCA) and in-hospital cardiac arrest (IHCA). However, cardiac arrests that occur in the emergency department (EDCA) could constitute a third category, due to the peculiar characteristics of the emergency department (ED). In recent years, the need to study EDCAs separately from other intra-hospital events has emerged. The aim of this study was to describe the characteristics and outcomes of a cohort of patients experiencing EDCA in an Italian hospital over a 14-year period. **Methods**: This was a single-centre retrospective observational study conducted in the ED of the Santa Croce e Carle Hospital in Cuneo, Italy. All adult patients who experienced EDCA between 1 January 2010 and 30 June 2023 were included. OHCA patients, those arriving in the ED with on-going resuscitation measures, patients with EDCA not undergoing resuscitation, and patients with post-traumatic cardiac arrest were excluded from the study. The main outcome of the study was survival at hospital discharge with a favourable neurological outcome. **Results**: 350 cases of EDCA were included. The median age was 78 (63–85) years, and the median Charlson Comorbidity Index score was 5 (3–6). A total of 35 patients (10%) survived to hospital discharge with a cerebral performance category (CPC) Score of 1–2; survival in the ED was 28.3%. The causes of cardiac arrests were identified in 212 cases (60.6%) and included coronary thrombosis (35%), hypoxia (22%), hypovolemia (17%), pulmonary embolism (11%), metabolic (8%), cardiac tamponade (4%), toxins (2%) and hypothermia (1%). Variables associated with survival with a favourable neurological outcome were young age, a lower Charlson Comorbidity Index, coronary thrombosis as the primary EDCA cause, and shockable presenting rhythm; however, only the latter was associated with the outcome in a multivariate age-weighted model. **Conclusions**: In a cohort of patients with EDCA over a period of more than a decade, the most frequent cause identified was coronary thrombosis; 10% of patients survived with a good neurological status, and the only factor associated with the best prognosis was presenting a shockable rhythm. EDCA should be considered an independent category in order to fully understand its characteristics and outcomes.

## 1. Introduction

Cardiac arrests (CA), defined as the sudden cessation of cardiac activity causing unresponsiveness with no signs of circulation or normal breathing [1], are traditionally classified according to the setting in which they occur, including out-of-hospital cardiac arrest (OHCA) and in-hospital cardiac arrest (IHCA) [2]. The incidence ranges from 92.3 individuals per 100,000 population for OHCA to 4.0 per 1000 hospitalisations for IHCA in the United States [3]. However, OHCA and IHCA differ not only epidemiologically but also in the clinical characteristics of patients, causes, survival rates and outcomes. Research has been conducted to improve cardiac arrest outcomes over the past two decades, and despite the optimisation of treatment algorithms and the training of healthcare professionals and laypersons in basic life support, mortality from CA remains high. The survival rate of IHCA is higher than that of OHCA [4], with patient survival to hospital discharge reported to be 35% and 14.6%, respectively [3,5]. Emergency Department Cardiac Arrest (EDCA) may comprise up to 19% of IHCA [6] and therefore could constitute a third category due to the peculiar characteristics of the emergency department (ED). These include a potentially higher proportion of reversible aetiologies, a lower burden of co-morbidity or conditions commonly associated with poor outcome (sepsis, malignancy, multiorgan dysfunction), and a higher potential for survival and better neurologic outcomes than IHCA (other locations than the ED) or OHCA patients conveyed to the ED [4,7,8,9,10]. Patients in the ED, however, may be more susceptible to CA due to less frequent assessments of vital records, crowding in the ED, and potential unstable patient conditions [11]. In recent years, the need to study EDCAs separately from other intra-hospital events has therefore emerged. The aims of this study were: (1) to describe a cohort of patients experiencing EDCA in an Italian hospital over a 14-year period, considering demographics, co-morbidities, cardiac arrest characteristics, and outcomes; and (2) to investigate early independent predictors of a good neurological outcome after EDCA.

## 2. Materials and Methods

### 2.1. Patients

This single-centre retrospective study was conducted in the ED at Santa Croce and Carle Hospital in Cuneo, a teaching hospital and a certified referral cardiac arrest centre with 75,000 visits per year in Piedmont, north-western Italy. All patients over 18 years of age with cardiac arrest occurring during their ED stay from 1 January 2010 to 30 June 2023 were included.

EDCA was defined as cardiac arrest occurring after a patient’s arrival in the ED and before discharge or admission to a ward. Patients with the following criteria were excluded from the study:

- Patients with OHCA, including patients arriving in the ED with on-going resuscitation;
- Patients who experienced cardiac arrest in the ED and were not undergoing resuscitation manoeuvres;
- Patients with post-traumatic cardiac arrest.

This study complied with the Declaration of Helsinki and was approved by the local ethics committee.

### 2.2. Outcomes

The main outcome was survival to discharge from hospital with a favourable neurological outcome, defined by a cerebral performance category (CPC) score of 1–2 [12]. The variables were collected through retrospective analysis of medical records by three emergency physicians blind to the outcome of the study. Data were collected and reported according to the Utstein style guidelines for reviewing, reporting, and conducting research on resuscitation and the updated and simplified Utstein templates for resuscitation registries [13,14].

Cardiac arrests were managed by emergency physicians and emergency nurses; all staff undergo training according to European Resuscitation Council guidelines for adult advanced life support, with updates every two years [15].

The comorbidity status of the patients was expressed by calculating the Charlson Comorbidity Index (CCI) [16]. The cause of the cardiac arrest was considered “identified” if reported by the emergency physician in the ED discharge diagnosis. In addition to the case history and the clinical presentation, the results of diagnostic tests completed in the ED were included: an electrocardiogram, an arterial blood gas test, a laboratory test (including electrolytes and cardiac enzymes), and X-rays. Point-of-care ultrasound was often performed as a useful instrument in ruling in or out the potential cause of cardiac arrest. A CT scan was performed, mainly before the cardiac arrest, and the results of the scan were documented as available. The causes of cardiac arrest were categorised according to the “4Is and 4Hs” approach proposed by the guidelines [15].

### 2.3. Statistics

Continuous variables with a normal distribution were presented as mean ± standard deviation, whereas those with a non-normal distribution were presented as median and interquartile range. Categorical variables were presented as frequencies and percentages. A univariate analysis was performed to identify significant differences between surviving patients with CPC 1–2 and other patients; subsequently, a multivariate model was obtained using the variables statistically significant in the univariate analysis. In all analyses, a *p* value < 0.05 was used for statistical significance. All statistical analyses were conducted using “R” software 4.0.0 GUI 1.71 Catalina build software (R Foundation for Statistical Computing, 2016).

## 3. Results

During the study period, 836,823 adult patients attended the ED (incidence 4/10,000). Among them, we identified 350 cases of EDCA according to the inclusion criteria (Table 1).

### 3.1. Patient Variables

The median age was 78 (63–85) years, and 59.7% were male, with a median CCI of 5 (3–6). In all, 84.8% of patients arrived at the ED using emergency medical services. A total of 38.3% of patients had known heart disease and 54% had arterial hypertension. In addition, 19.7% of patients had diabetes, 14% had chronic lung disease, and 11% of patients had chronic kidney disease. Also, 8.8% of patients had cancer and 3% had a history of substance abuse.

### 3.2. Cardiac Arrest Variables

A total of 14.3% of patients had a shockable rhythm at the onset of cardiac arrest (10.9% ventricular fibrillation and 3.4% pulseless ventricular tachycardia); the most frequent presenting rhythm was pulseless electrical activity (63.1%) (Table 2). A total of 43.4% of patients underwent orotracheal intubation. Resuscitation manoeuvres were performed for a median of 15 [4;30] minutes; in patients with a positive outcome, resuscitation manoeuvres lasted a median of 15 [7,8,9,10,11,12,13,14,15,16,17,18,19,20] minutes vs. 18 [8,9,10,11,12,13,14,15,16,17,18,19,20,21,22,23,24,25,26,27,28,29,30] in patients with a negative outcome (not a statistically significant difference). Adrenaline was administered in 280 cases (80%), amiodarone in 30 cases (15%), and lidocaine was not administered in any patients. The endotracheal tube was the only device used for airway management in 332 cases (92%). The cause of cardiac arrest was identified in 212 cases (60.6% of all patients). The most frequent cause was coronary thrombosis (34.9%), followed by hypoxia (22.2%), hypovolemia (17.4%), pulmonary embolism (10.9%), metabolic causes/hypokalaemia/hyperkalaemia (8.5%), cardiac tamponade (3.7%), toxins (1.9%), and hypothermia (0.5%) (Table 3). Specifically, in cases of coronary thrombosis, percutaneous coronary intervention (PCI) was performed in 34 cases (46%); and coronary artery bypass graft surgery (CABG) in 3 cases (2%). Thrombolysis was performed with alteplase in all cases of pulmonary embolism.

### 3.3. Outcome Variables

In 107 (30.6%) patients, ROSC was obtained in the ED, and survival at hospital admission after ED treatment was 28.3%. Overall survival at 30 days was 15.1%; 35 patients (10%) survived to hospital discharge with a CPC score of 1–2 (Table 4).

Patients who survived at hospital discharge with CPC score of 1–2 had a shockable rhythm at presentation in 54.3% of cases, vs. 9.8% of other patients (*p* < 0.001); furthermore, they were younger (61 [46;73] years vs. 79 [65;85] years, *p* < 0.001), had a lower CCI score (3 [2;5] vs. 5 [3;6], *p* < 0.001), and more frequently the cause of cardiac arrest was cardiac (54.3% vs. 18.4%, *p* < 0.001) (Table 1). In the multivariable analysis, only shockable rhythm was associated with a favourable outcome in an age-weighted model (Table 5).

## 4. Discussion

In this 14-year retrospective study of patients who experienced cardiac arrest during ED stay, the majority of patients were older males with known heart disease. The most frequent presenting rhythm was pulseless electrical activity, and the most frequent cause of CA was coronary thrombosis. Survival at hospital admission after ED treatment was 28.3%, and overall survival at 30 days was 15.1%. A good long-term neurological outcome (CPC1–2) was recorded in only 10% of patients who survived to hospital discharge. On multivariable analysis, only shockable rhythm was associated with a favourable outcome in an age-weighted model. In accordance with a previous study completed by Van Walraven et al., which shows a higher mortality rate for resuscitation attempts longer than 10 minutes, our data also suggests that a longer period of resuscitation could be associated with worse outcomes [17,18].

Studies that have previously studied EDCA varied in population size and in definitions of cohorts (e.g., CA in patients with traumatic CA), which may have impacted the characteristics and outcomes. In Kayser et al.’s study [7] of over 60,000 patients with IHCA, 7435 had EDCA (including patients with traumatic CA), of which the mean age was 63.8 years, 59.3% were male; the ED had the largest percentage of patients with no known pre-existing conditions and the lowest percentage of patients with sepsis, pneumonia, acute stroke, malignancy, diabetes, hepatic insufficiency, renal insufficiency, and heart failure than those suffering a CA in other locations (e.g., ICU, floor). ED patients had the highest percentage of individuals with acute myocardial infarction, pulmonary oedema, or a toxicological condition as a predisposing cause. Similar to this study’s findings, PEA was the most frequently observed first pulseless rhythm, although ED patients had a higher prevalence of ventricular fibrillation and a lower prevalence of asystole as the initial rhythm compared to other in-hospital patients. Survived to discharge for EDCA patients was 22.8%, higher than our data, with a significantly higher survival to discharge percentage than other in-patients. ED discharge CPC values were significantly better compared to other locations.

More recently, Mir and Colleagues [19] analysed a total of 1,068,847 cardiac arrests in the Nationwide Emergency Department Sample (NEDS) between 2016 and 2018. A total of 325,062 (30.4%) cardiac arrests occurred in the ED. As in the previous study, EDCA had a lower burden of comorbidities than IHCA. The predominant causes associated with EDCA were trauma (6.4%), followed by intoxication (7.5%), respiratory failure (5%), and ST-elevated myocardial infarction (2.5%), with an unknown diagnosis reported in the majority of cases, as a larger proportion of the patients did not survive. It appears that fewer comorbidities do not correspond to lower mortality; of the patients who had EDCA, 10% survived post-CPR until hospital discharge, compared to 31.6% of IHCA patients. In contrast, in this study among EDCA patients, traumatic CA and first cardiac arrest outside the hospital were also included. Survival in our study is relatively lower compared to other studies that have examined EDCA, in which a survival rate at discharge of between 18.8% and 20.43% was reported [20,21]. We did, however, analyse the survival at 30 days and not at discharge, and this may have impacted the results. The low survival rate with a positive neurological outcome, compared to what has been reported in other studies, can be explained by the significantly advanced age of our patient cohort and by the number of comorbidities. The low survival rate in the ED may also be explained in this way. Furthermore, the low incidence of shockable rhythms could be implicated in the low occurrence of ROSC. Nevertheless, the survival rate for EDCA is highly variable in the few studies available in the literature, and our data are comparable to other larger series [19].

The observation that patients with shockable rhythms present significantly better outcomes could suggest that patients identified as at greater risk of deterioration should be subjected to continuous ECG monitoring, even in the ED, in order to intercept any cardiac arrests as early as possible [22]. As expected, in our study, patients with fewer comorbidities had higher survival rates, and patients who survived with a CPC score of 1–2 had a lower CCI score. This association, however, has not been demonstrated in other cohorts. In a study conducted in the EDs of the United States, there was a negative association between conventional risk factors and mortality, with the exception of a history of prior myocardial infarction [19]. Chen et al. also found a statistically significant difference in CCI between survivor and non-survivor patients [20].

In our multivariable analysis, shockable presentation rhythm was associated with a favourable outcome in an age-weighted model; 54.3% of patients survived with a good neurological outcome presented with VF or VT as compared with 9.8% of other patients (who died or survived with a poorer neurological outcome). These data are consistent with those of the Swedish Registry for Cardio-Pulmonary Resuscitation concerning EDCA between 2007 and 2018 [23], in which 50% of survivors and 13% of non-survived patients presented with a shockable rhythm. Percentages of initial rhythm actually vary among different patients’ cohorts; nevertheless, an initial shockable rhythm has almost always shown a more favourable survival rate and better CPC at discharge [19,21,24].

EDCA could be considered a third and distinct category of cardiac arrest, with a survival rate that is lower than that of IHCA (according to the GWTG-R, survival to hospital discharge is 25% [25,26]) but higher than that of OHCA (varying from 3% in Asia to 9.7% in Australia [27]). This difference in survival rate has a number of explanations. In OHCA, the number of witnessed arrests is lower if compared to IHCA and EDCA, and, moreover, CPR is not always started immediately [4,28]; the bystander CPR rate is still variable (from 13% to 82%, with an average of 58%, among European countries [29]). The delay in commencing CPR can partially justify the higher mortality rate. The situation of IHCA is even more changeable since the percentage of witnessed CA and shockable rhythm as presenting rhythm varies from the ICU to other wards [7].

Other differences include the frequency of aetiologies. A meta-analysis published in 2022 by Allencherril et al. demonstrated that the most frequent cause of IHCA was hypoxia (26.5% vs. 22.2% in our study), followed by acute coronary syndrome (18.2% vs. 34.9% among our patients) [30,31]. Among OHCA, traumatic causes have an important role, with an average rate of 3.9%. [29]

As previous studies demonstrate that EDCA represents an important condition with a poorer prognosis if compared with IHCA, predicting imminent CA in the ED would be beneficial. This difference between EDCA and IHCA could be, at least partially, explained by the availability of closer monitoring of admitted patients. Moreover, it could also be easier to identify the cause of cardiac arrest in hospitalised patients since the whole medical history is already known by the medical team. Early warning scores could play an essential role in identifying patients with a high risk of cardiac arrest in order to provide them with intensive monitoring and guarantee an early intervention. Recently, Tsai et al. [32] developed and validated a predictive tool to define the risk of having a cardiac arrest in an ED. The score contains the following eight items: age, mean of arrival at the hospital, systolic blood pressure, heart rate, body temperature, respiratory rate, oxygen saturation, GCS, or acute change in level of consciousness. A score greater than 6 corresponds with a high risk. This tool has also been externally validated in a retrospective cohort study conducted in 2022 in Taiwan [33].

Srivilaithon et al. also analysed predictors of cardiac arrest within 24 hours of ED admission [34]. They suggest that using NEWS, a widely known and used score [35], in combination with monitoring its change at different times might help in identifying patients at risk of early cardiac arrest. Nevertheless, a combination of NEWS and other clinical predictors showed a greater ability to predict the risk. These predictors are coronary artery disease, initial serum bicarbonate level, and use of vasoactive agents.

Another tool that could be implemented in order to predict cardiac arrest in the ED is machine learning. Despite the impressive performance of these learning scores, studies about their use on this topic are still lacking [8]. In 2012, Ong and colleagues developed a machine aiming to predict cardiac arrest within 72 hours after triage; this instrument appeared to perform better than other early warning scores, but it was validated only on patients requiring continuous ECG monitoring [36]. Some years later, another retrospective cohort study was conducted, collecting data on ED visits over 7-year period in Taiwan. Three machine learning models (Random Forest, Gradient Boosting, and Extra Trees) were validated and showed very good discriminatory performance to identify EDCA using only triage information; all of them actually performed significantly better than the NEWS2 scoring system. These models appeared to have excellent discriminatory performance and very high specificity. These tools are constructed using only clinical features readily available at ED triage, including consciousness, age, triage level, vital-sign parameters (particularly respiratory rate, oxygen saturation, pulse rate, and systolic blood pressure), and various clinical symptoms such as chest pain, dyspnoea, and injury. Such models could therefore potentially help identify high-risk patients and prevent unexpected in-hospital deaths [37].

A quite recent study analysed the association between ED overcrowding and unexpected cardiac arrest, showing that higher ED occupancy rates were strongly associated with sudden IHCA occurrence [38]. Besides crowding, the occurrence of CA is also influenced also by the proportion of critically ill patients. Despite this, ED occupancy was found not to be correlated with ED mortality. 

As previously stated, all our clinicians have been trained according to European Resuscitation Council guidelines for adult advanced life support, which have been demonstrated to improve patient outcomes [39,40].

This study has some limitations. It was a retrospective study with a small sample size, in which all the information was only derived from patients’ medical records. As in OHCA and many IHCA studies, even if all CA were witnessed, not all patients were continuously monitored, meaning it is not possible to be sure about the initial arrest rhythm.

## 5. Conclusions

In a cohort of patients with EDCA over a period of more than a decade, the most frequent cause of cardiac arrest was coronary thrombosis, and the most frequent presenting rhythm was PEA. It may be necessary that EDCA be studied as a distinct category in order to fully understand its characteristics and improve patients’ outcomes, which are known to be poor. Furthermore, the use of tools such as scores or machine learning in order to predict imminent CA in the ED would be beneficial to identify high-risk patients and prevent unexpected deaths, particularly in an overcrowded ED.

## Figures and Tables

**Table 1:** Patient and cardiac arrest characteristics.

	Sample Total (n = 350)	Survived with CPC 1–2 Patients (n = 35)	Other Patients (n = 315)	*p* Value
Age	78 [73;85]	61 [46;73]	79 [65;85]	<0.001
Male	209 (59.7%)	26 (74.3%)	183 (58.1%)	0.06
Charlson index	5 [3;6]	3 [2;5]	5 [3;6]	<0.001
Arrived via EMS	297 (84.8%)	27 (77.1%)	270 (85.7%)	0.17
Cardiopathy	134 (38.3%)	14 (40%)	120 (38.1%)	0.91
Shockable presentation rhythm	50 (14.3%)	19 (54.3%)	31 (9.8%)	<0.001
Cardiac CA cause	77 (22%)	19 (54.3%)	58 (18.4%)	<0.001

Continuous variables are presented as medians [25–75th]; categorical variables are presented as frequencies and percentages. CPC, cerebral performance category; EMS, emergency medical services; CA, cardiac arrest.

**Table 2:** Presentation rhythm, [n, (%)].

Presentation Rhythm	
Pulseless cardiac activity	221 (63.1)
Asystole	79 (22.6)
Ventricular fibrillation	38 (10.9)
Ventricular tachycardia	12 (3.4)

**Table 3:** Identified cardiac arrest causes, [n, (%)].

Cause	n = 212
Coronary thrombosis	74 (34.9)
Hypoxia	47 (22.2)
Hypovolemia	37 (17.4)
Pulmonary embolism	23 (10.9)
Metabolic	18 (8.5)
Cardiac tamponade	8 (3.7)
Toxins	4 (1.9)
Hypothermia	1 (0.5)

Variables are presented as frequencies and percentages.

**Table 4:** Patients outcomes, [n, (%)].

Outcomes	
ROSC in ED	107 (30.6)
ED survival	99 (28.3)
30 day survival	53 (15.1)
30 day survival with CPC 1–2	35 (10)

ROSC, return of spontaneous circulation; ED, emergency department; CPC, cerebral performance category.

**Table 5:** Multivariate analysis.

	Odds Ratio	95% C.I.	*p* Value
Shockable presentation rhythm	12.23	3.42–31.93	<0.001
Age	0.97	0.95–0.98	0.019

## Data Availability

The authors confirm that the data supporting this manuscript are available within the article.
